# Resuscitation in Paediatric Sepsis Using Metabolic Resuscitation–A Randomized Controlled Pilot Study in the Paediatric Intensive Care Unit (RESPOND PICU): Study Protocol and Analysis Plan

**DOI:** 10.3389/fped.2021.663435

**Published:** 2021-04-30

**Authors:** Luregn J. Schlapbach, Kristen Gibbons, Roberta Ridolfi, Amanda Harley, Michele Cree, Debbie Long, David Buckley, Simon Erickson, Marino Festa, Shane George, Megan King, Puneet Singh, Sainath Raman, Rinaldo Bellomo, The RESPOND PICU study investigators and the Australian New Zealand Intensive Care Society Paediatric Study Group (ANZICS PSG)

**Affiliations:** ^1^Child Health Research Centre, The University of Queensland, and Paediatric Intensive Care Unit, Queensland Children's Hospital, Brisbane, QLD, Australia; ^2^Pediatric and Neonatal Intensive Care Unit, and Children's Research Center, University Children's Hospital Zurich, Zurich, Switzerland; ^3^Departments of Emergency Medicine and Children's Critical Care, Gold Coast University Hospital, Southport, QLD, Australia; ^4^School of Nursing, Midwifery and Social Work, University of Queensland, Brisbane, QLD, Australia; ^5^Pharmacy Department, Queensland Children's Hospital, Brisbane, QLD, Australia; ^6^School of Nursing, Centre for Healthcare Transformation, Queensland University of Technology, Brisbane, QLD, Australia; ^7^Paediatric Intensive Care Unit, Starship Children's Hospital, Auckland, New Zealand; ^8^Paediatric Critical Care Unit, Perth Children's Hospital, Perth, WA, Australia; ^9^Paediatric Intensive Care Unit, Children's Hospital Westmead, Sydney, NSW, Australia; ^10^Kids Critical Care Research Group, Kids Research, Sydney Children's Hospitals Network, Sydney, NSW, Australia; ^11^School of Medicine and Menzies Health Institute Queensland, Griffith University, Southport, QLD, Australia; ^12^Paediatric Intensive Care Unit, Sydney Children's Hospital, Sydney, NSW, Australia; ^13^Intensive Care Research, Austin Hospital and Monash University, Melbourne, VIC, Australia

**Keywords:** ascorbic acid, child, hydrocortisone, intensive care, sepsis, septic shock, thiamine, vitamin C

## Abstract

**Introduction:** Septic shock remains amongst the leading causes of childhood mortality. Therapeutic options to support children with septic shock refractory to initial resuscitation with fluids and inotropes are limited. Recently, the combination of intravenous hydrocortisone with high dose ascorbic acid and thiamine (HAT therapy), postulated to reduce sepsis-related organ dysfunction, has been proposed as a safe approach with potential for mortality benefit, but randomized trials in paediatric patients are lacking. We hypothesize that protocolised early use of HAT therapy (“metabolic resuscitation”) in children with septic shock is feasible and will lead to earlier resolution of organ dysfunction. Here, we describe the protocol of the Resuscitation in Paediatric Sepsis Using Metabolic Resuscitation–A Randomized Controlled Pilot Study in the Paediatric Intensive Care Unit (RESPOND PICU).

**Methods and Analysis:** The RESPOND PICU study is an open label randomized-controlled, two-sided multicentre pilot study conducted in paediatric intensive care units (PICUs) in Australia and New Zealand. Sixty children aged between 28 days and 18 years treated with inotropes for presumed septic shock will be randomized in a 1:1 ratio to either metabolic resuscitation (1 mg/kg hydrocortisone q6h, 30 mg/kg ascorbic acid q6h, 4 mg/kg thiamine q12h) or standard septic shock management. Main outcomes include feasibility of the study protocol and survival free of organ dysfunction censored at 28 days. The study cohort will be followed up at 28-days and 6-months post enrolment to assess neurodevelopment, quality of life and functional status. Biobanking will allow ancillary studies on sepsis biomarkers.

**Ethics and Dissemination:** The study received ethical clearance from Children's Health Queensland Human Research Ethics Committee (HREC/18/QCHQ/49168) and commenced enrolment on June 12th, 2019. The primary study findings will be submitted for publication in a peer-reviewed journal.

**Trial Registration:** Australian and New Zealand Clinical Trials Registry (ACTRN12619000829112). **Protocol Version**: V1.8 22/7/20.

## Introduction

Sepsis, defined as dysregulated host response to infection leading to life-threatening organ dysfunction, ranks amongst the leading causes of childhood mortality (1–3). Sepsis remains responsible for an estimated three million childhood deaths each year worldwide (4, 5). Despite advances in paediatric intensive care practices, the mortality due to septic shock remains high (around 17%) (6–8). Paediatric sepsis survivors often suffer from long-term sequelae, resulting in a lifelong burden to patients, families, and the society (9, 10).

Paediatric septic shock often is a fulminant disease, and affected children deteriorate rapidly (11). Hence, in order to optimize outcomes and prevent irreversible multi-organ damage, future interventions will need to be applied early after presentation. Therapeutic options for children where septic shock persists despite initial resuscitation remain scarce. Current recommendations for treatment of refractory paediatric septic shock include consideration for intravenous hydrocortisone (12). Several large randomized-controlled trials in septic adults have demonstrated earlier reversal of septic shock using treatment with hydrocortisone (13–15). In children, guidelines explicitly state that clinical trial evidence for the role of hydrocortisone in paediatric septic shock remains unclear, however the use of hydrocortisone appears to be very common (16–18). More recently, ascorbic acid (Vitamin C), has been proposed as a sepsis adjunct therapy. Ascorbic acid carries powerful antioxidant properties and *in-vitro* and animal model evidence indicates protective effects on endotoxin-related endothelial damage and organ function (19, 20). Given that thiamine deficiency is associated with high lactate (21) and an increased risk of death (21), a protocol combining hydrocortisone, ascorbic acid, and thiamine (HAT therapy, or so called metabolic resuscitation) has been trialled in adult studies with some reporting mortality benefit (22, 23), while other trials did not observe differences in patient-centred outcomes (24–28). Both thiamine and ascorbic acid carry an excellent safety profile (29). A recent systematic review on metabolic resuscitation (30) identified nine registered RCTs on metabolic resuscitation in adult septic shock, but none in children. Children may be more prone to vitamin C deficiency during sepsis due to comorbidities, malnutrition, a higher metabolic rate, and a higher proportion of patients with a rapidly progressive disease course. A propensity-matched cohort study from the USA reported excellent safety and feasibility of metabolic resuscitation in children with septic shock (31). Adjusted analyses showed decreased 30- and 90-day mortality (*p* < 0.05) in the intervention group compared to hydrocortisone alone and compared to standard care without hydrocortisone.

We therefore designed the pragmatic Resuscitation in Paediatric Sepsis Using Metabolic Resuscitation–A Randomized Controlled Pilot Study in the Paediatric Intensive Care Unit (RESPOND PICU) to test the feasibility of a paediatric RCT comparing metabolic resuscitation vs. standard septic shock management in children aged ≥28 days to <18 years requiring inotropes for suspected septic shock. We hypothesize that a protocol on metabolic resuscitation in children is feasible and that the intervention will lead to faster resolution of shock compared to standard shock management. In this paper, we describe the RESPOND PICU study protocol including the statistical analysis plan.

## Methods

### Study Design and Setting

**Figure 1:**
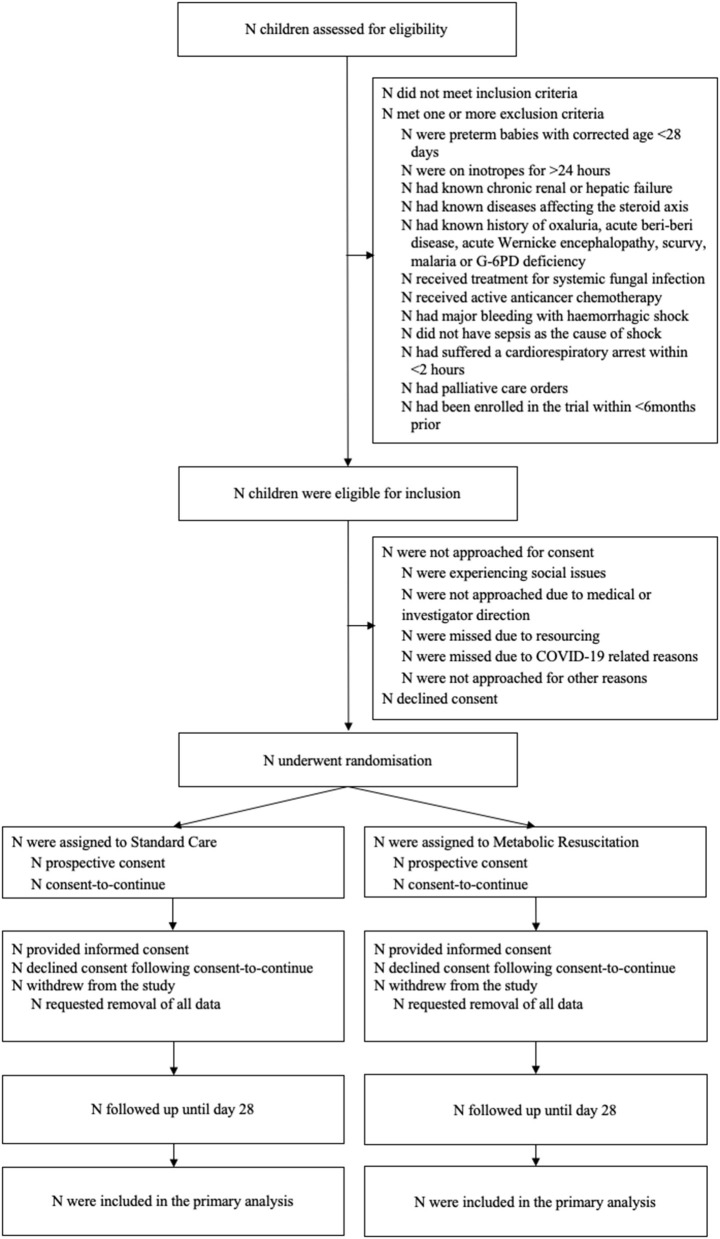
Draft CONSORT participant flow diagram for RESPOND PICU.

The RESPOND PICU study is an open label, multicentre, pragmatic randomized controlled pilot trial (RCT) for children aged between 28 days and 18 years who are admitted to PICUs requiring inotrope therapy for presumed septic shock (Figure 1). The study will recruit patients in tertiary PICUs of participating sites in Australia and New Zealand. The trial compares ***metabolic resuscitation*, **defined as hydrocortisone, high dose ascorbic acid, and thiamine, with ***standard care***defined as septic shock management according to institutional protocols (12, 32). The study protocol has been approved by the Children's Health Queensland Hospital and Health Service Human Research Ethics Committee (HREC/18/QCHQ/49168) and is registered with the Australian New Zealand Clinical Trials Registry (ACTRN12619000829112).

### Participants

#### Inclusion Criteria

Children aged between 28 days and 18 years admitted to PICU and receiving inotrope therapy for septic shock for at least 2 h are eligible (Table 1). Inotropes (and vasopressors) include adrenaline, noradrenaline, vasopressin, milrinone, dopamine, and dobutamine. Patients treated with inotropes, independent on the dose or route of administration are eligible. Children not meeting age criteria, those with chronic liver failure, cardiomyopathy, those with conditions affecting steroid or vitamin metabolism, and children under active chemotherapy will be excluded. In addition, children with palliative care orders, and children who suffered a cardiorespiratory arrest in the 2 h preceding enrolment are excluded.

**Table 1:** Inclusion and Exclusion criteria.

**Rule**	**Criterium**	**Definition**
Inclusion	Age	• Aged ≥28 days and <18 years
	Illness	• Admitted to PICU and treated for septic shock
	Treatment	• Received inotropes for at least 2 h
	Consent	• Parental/caregiver consent prior to or after enrolment
Exclusion	Age	• Preterm babies born <34 weeks gestation that have a corrected age of <28 days
	Treatment	• Received inotropes for >24 h pre-enrolment • Patient is receiving treatment for systemic fungal infection or has documented strongyloides infection at the time of randomization • Patient undergoing active chemotherapy for cancer treatment
	Co-morbidities	• Cardiomyopathy (not due to sepsis) or chronic cardiac failure • Chronic hypertension due to cardiovascular or renal disease, requiring regular antihypertensive treatment. • Known chronic renal failure (defined as requiring Renal Replacement Therapy) • Known chronic hepatic failure (defined as pediatric Sequential Organ Failure Assessment hepatic subscore >0) • Known diseases affecting the steroid axis, including pituitary disease, congenital adrenal hypoplasia, Cushing or Addison's disease • Known glucose-6 phosphate dehydrogenase (G-6PD) deficiency • Patients with known history of oxalate nephropathy • Patients with acute beri-beri disease • Patients with acute Wernike's encephalopathy • Patients with known malaria • Patients with known of suspected scurvy • Palliative care patient/patient with limitation of treatment (not for inotropes, cardiopulmonary resuscitation, extracorporeal membrane oxygenation, intubation or ventilation)
	Illness severity	• Cardiopulmonary arrest in the past 2 h requiring cardiopulmonary resuscitation of >2 min duration, or death is deemed to be imminent or inevitable during this admission. • Major bleeding with haemorrhagic shock • Sepsis is not likely to be the cause of shock
	Previous study enrolment	Enrolment in RESPOND study <6 months prior

The pragmatic protocol stipulates the use of inotropes as the main feature of shock and does not mandate specific vital signs or laboratory thresholds to qualify for shock.

#### Recruitment

**Figure 2:**
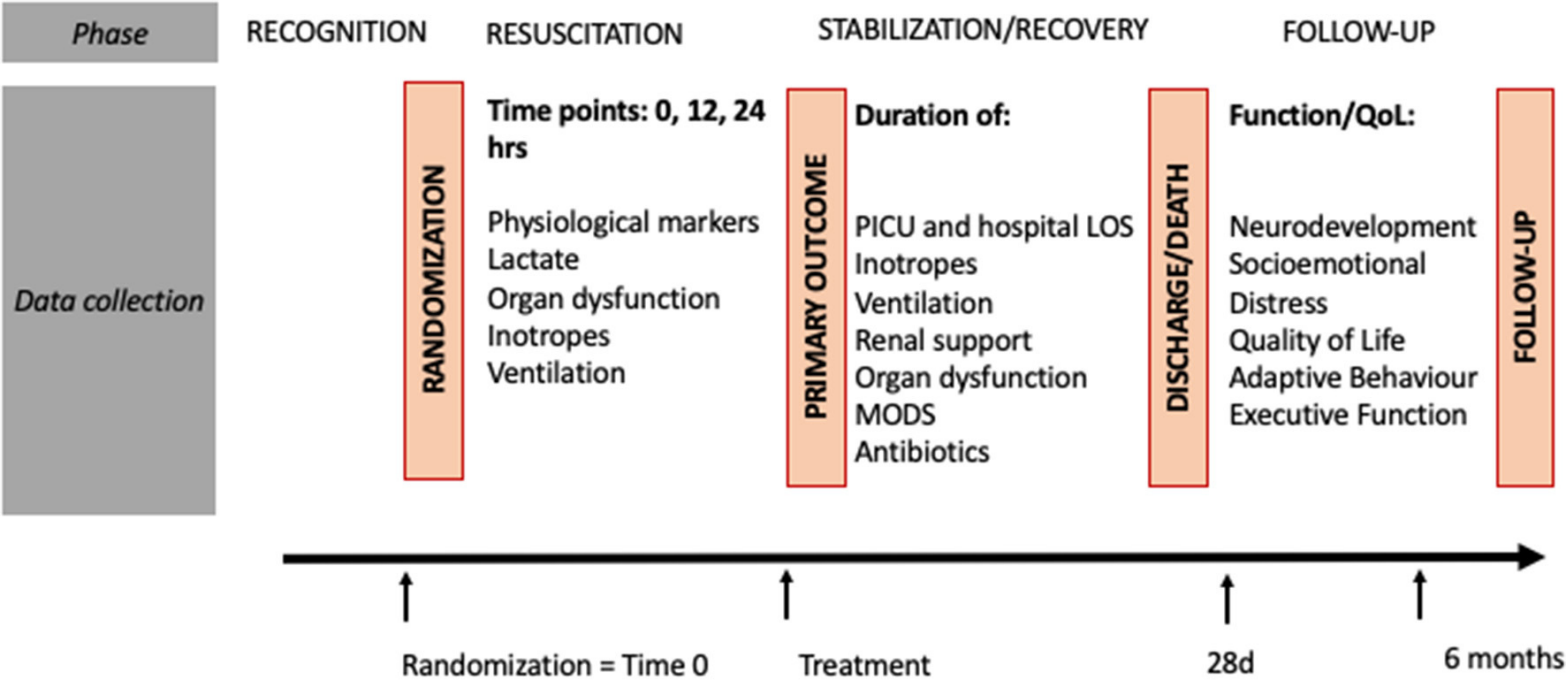
Study procedures. ASQ, ages and stages; BRIEF, paediatric Behavior Rating Inventory of Executive Function; FSS, functional status score; LOS, length of stay; MODS, multiple organ dysfunction syndrome; PICU, paediatric intensive care unit; POPC, Paediatric Overall Performance Category; QoL, quality of life.

Screening and recruitment of participants will occur in the PICU (Figure 1). Study staff will approach the parents or guardians for prospective consent where feasible. Consent will include permission to conduct a follow-up assessment by questionnaire at 6 months post randomization (Figure 2). Separate consent will be sought to allow biobanking for ancillary studies on sepsis biomarkers. If timely informed consent is not feasible (for example if parents are not present immediately or if they are too distressed) the study team can employ consent to continue (33), until parents can be approached to seek written consent. Parents have the option to withdraw consent, which will lead to their child's study data being excluded.

### Randomization and Blinding

Allocation occurs in a 1:1 ratio to the treatment group (receiving metabolic resuscitation vs. standard shock management). We will use a permuted block randomization with variable block sizes of two, four and six stratified by site. Sealed opaque envelopes will be provided using a randomization sequence generated by The University of Queensland, Brisbane, Australia. Once a child meets eligibility criteria, study staff or treating clinicians are advised to open the next sequential sealed envelope to reveal the allocation of the patient.

Due to the challenges in blinding three drugs, and considering the main aim of this study to investigate feasibility of metabolic resuscitation, the study will be open label.

### Study Interventions

The study treatment should be started immediately after randomization. The RCT compares ***metabolic resuscitation***with ***standard care***.

#### Metabolic Resuscitation

After initial shock treatment including fluids and intravenous inotrope(s), patients then receive additional treatment with hydrocortisone, ascorbic acid, and thiamine:

#### Ascorbic Acid (Vitamin C)

The dosing schedule is 30 mg/kg/dose (maximum 1,500 mg per dose) intravenously every 6 h for the duration of study treatment and will be infused over 30–60 min.

#### Thiamine (Vitamin B1)

The dosing schedule is 4 mg/kg/dose (maximum 200 mg per dose) intravenously every 12 h for the duration of study treatment and will be infused over 30–60 min.

#### Hydrocortisone

The dosing schedule is 1 mg/kg/dose (maximum 50 mg per dose) intravenously every 6 h for the duration of study treatment given as a slow bolus.

Study drugs will be given through an existing peripheral or central intravenous line. Drug delivery will occur through dose error reduction software for infusion devices to ensure safe delivery of applied standardized drug concentrations. The dosing is aligned with the ADRENAL steroid treatment regimen (13), and with the protocol for the VITAMIN trial (24) and has been shown to restore serum ascorbic acid concentrations in critically ill patients (34). The study treatment will be given for a maximum of 7 days, until resolution of shock (defined as cessation of inotropes for at least 4 h), discharge from PICU, death, or if major adverse events related to the intervention manifest, whichever occurs first.

#### Standard Care

Patients in the standard treatment arm can receive hydrocortisone only if clinically indicated at the discretion of the attending ICU staff specialist and should be treated as per the standard institutional approach to septic shock management (12, 32).

#### Other PICU Care

The study protocol does not prescribe what type, dose, or combination of inotropes should be used. Fluid boluses in all three arms can be balanced or unbalanced crystalloid or colloid fluids. Other care including respiratory management, antimicrobials, glucose and electrolyte control, transfusion, sedation, and extracorporeal life support should be provided at the discretion of the treating physician according to local practice.

### Study Outcomes

In addition to feasibility outcomes, the study will assess clinical outcome measures, and proxy measures of intervention efficacy (Table 2). *Feasibility outcomes* include consent rates, time to intervention, hydrocortisone administration prior to randomization, and post randomization in the control group, and protocol violations. Survival free of organ dysfunction censored at 28 days is defined as the *primary clinical outcome*. The pediatric Sequential Organ Failure Assessment (pSOFA) score will be used to assess presence and degree of organ dysfunction (36). All-cause deaths occurring within 28 days of enrolment will be allocated zero organ dysfunction free days. pSOFA scores will be assessed every day during which a patient is admitted in PICU. In children not requiring ongoing respiratory or renal support post discharge from PICU, and not under palliative care, those discharged from PICU will be assumed to have no organ dysfunction which is aligned with the practice at the participating institutions. PICU free survival, survival free of inotrope support, survival free of multi-organ dysfunction, survival free of acute kidney injury (AKI) (37), mortality, PICU and hospital length of stay represent the *secondary clinical outcome measures*. In addition, study nurses will contact families at 28 days to perform a Pediatric Overall Performance Category (POPC) (38) and Functional Status Score (FSS) assessment (39). As a modification from the POPC “good” category (healthy, alert, and capable of normal age-appropriate activities of daily life; medical and physical problems do not interfere with normal activity), we divided the first POPC category into “good/normal” (no medical conditions), and b) “functionally normal” (requires medication and medical input, normal intellectually and physically, able to do activities without restriction).

**Table 2:** Outcomes assessed.

**Outcome**	**Criterium**	**Definition**
Feasibility of the protocol	Recruitment and compliance with study protocol	• Recruitment rates; including (i) proportion of eligible randomized, and (ii) proportion of eligible consented using prospective consent and consent to continue. • Time to initiation of metabolic resuscitation in the intervention arm • Proportion of patients in each study arm which receive hydrocortisone prior to enrolment • Proportion of patients in the control arm which receive hydrocortisone after enrolment • Protocol violations
Primary clinical outcome	Survival free of organ dysfunction	• Organ dysfunction defined as pediatric Sequential Organ Failure Assessment (pSOFA) score >0, and will be assessed daily until day 28 while patients are admitted to PICU. Patients dying within 28 days of presentation will be allocated zero days to correct for the competing effect of mortality on duration of organ dysfunction. Patients discharged alive from PICU to the ward or home not requiring ongoing organ support, and not being palliative, will be assumed to have zero organ dysfunction after PICU discharge.
Secondary clinical outcomes	Patient-centred outcomes	• Survival free of inotrope support at 7 days • Survival free of multiorgan dysfunction at 7 days
		• 28-day mortality • Survival free of PICU censored at 28 days • Survival free of AKI censored at 28 days^*^ • PICU length of stay • Hospital length of stay • Functional Status Score and modified Pediatric Overall Performance Category at 28 days • Neurodevelopment, Quality of Life and Functional status 6 months post enrolment
	Proxy measures of intervention efficacy	• Proportion with serum lactate <2 mmol/l at 6, 12, and 24 h post enrolment • Time to normalization of tachycardia during the first 24 h post enrolment^**^ • Time to shock reversal, defined as cessation of inotropes for at least 4 h censored at 28 days

* *Acute Kidney Injury (AKI) will be assessed using serum creatinine levels to classify according to Kidney Disease: Improving Global Outcomes (KDIGO) criteria. Because no baseline creatinine values were available, we applied the age-specific thresholds used in PELOD-2 to define the presumed baseline creatinine values. KDIGO Stage 1 was defined as an increase in creatinine to 1.5 to 1.9 times the presumed baseline; Stage 2 as an increase 2.0 to 2.9 times; and KDIGO 3 as an increase ≥3.0 baseline and/or the use of renal replacement therapy*.

** *Goldstein et al. (35)*.

Long-term follow-up at 6 months post randomization through questionnaire by proxy on validated domains across quality of life (40) and functional status will be investigated and reported separately from the main analyses.

Furthermore, we will determine *proxy measures of intervention efficacy* such as shock reversal, normalization of lactate, and time to reversal of tachycardia [defined by age-specific thresholds for Systemic Inflammatory Response Syndrome (35)].

### Adverse Events (AEs)

Based on the anticipated severity of the study population informed by recent observational studies in Australian and New Zealand PICUs (7, 11, 41) a proportion of study patients are expected to suffer from complications related to septic shock unrelated to study interventions (42). Major AEs including death, cardiopulmonary arrest, ECMO and amputations will be captured in all patients. In addition, we will report any AE potentially causally related to the study intervention or which is of concern in the investigator's judgement. Specific additional AEs captured include limb ischemia, extravasation injury, hypertension, arrhythmia other than sinus bradycardia or tachycardia, hyperglycaemia, abdominal compartment syndrome, pulmonary edema and confirmed hospital-acquired infection. Adverse events will be assessed routinely until day 28, or until the time of patient discharge from hospital, whichever occurs earlier. The *Data and Safety Monitoring Board (DSMB)* consists of a statistician, an ICU specialist and an emergency specialist will receive regular DSMB reports and immediate access to any serious adverse event reports. The DSMB, once set up, will meet mid-trial and at the completion of the trial.

### Data Collection

The central study coordination will provide a study booklet for site staff, and perform site visits and regular videoconferencing to ensure high standard study conduct and to discuss any challenges in study progress. The REDCap online database (43), hosted by The University of Queensland, has been setup to capture baseline demographic variables, comorbidities, disease and severity features, pre-defined study outcomes, information on study treatments, and adverse events. The staff at each study site will be trained in completing this electronic case report form (eCRF) and in performing the follow-up questionnaires. Study participants alive at day 28 will be contacted by phone (unless still in hospital) to assess POPC and FSS. Subsequently, the study nurses will contact families ~2 months post randomization by phone to perform the questionnaires by proxy on neurodevelopment, quality of life and functional outcomes–details of the follow-up will be published separately.

Physiological parameters and organ support will be collected upon randomization, then at 1, 6, 12, and 24 h subsequently. We will collect individual components of organ dysfunction and organ support every day until discharged from PICU to calculate pSOFA scores.

### Biobanking

Where parental consent is available, we will obtain 1–2 mL of EDTA blood, 2.5 mL of PAXgene blood, and 1–2 mL of serum as close to enrolment as possible. The samples will be transferred to a biobank using standard operating procedures and will remain stored for anxillary studies on sepsis markers.

### Data Quality and Monitoring

A detailed monitoring plan has been established which guides the data validity checks embedded in REDCap and monitoring by an auditor not involved in entering the study data. Specifically, we will perform primary source data verification in 100% of enrolled patients for key variables such as randomization allocation, commencement of intervention and consent, study treatments, organ support, PICU length of stay, survival status, and reported protocol deviations and adverse events. Furthermore, we will randomly select 10% of patients where a range of other study data will be verified using primary source verification, such as inclusion and exclusion criteria for ineligible patients, baseline data, first 24 h data, demographic data, daily organ dysfunction data, and hospital discharge data. Regular training of study staff and videoconferences with the central study team will be held to observe study conduct.

### Statistical Analysis Plan

#### Sample Size

We define feasibility of recruitment if ≥65% of eligible patients are enrolled. Enrolment of 60 of 80 eligible patients will yield a recruitment rate of 75% with a one-sided lower 95% confidence interval limit of 66%, which would meet our feasibility metric for recruitment rate. We anticipate a recruitment period of 24 months to meet the target sample size of 60 participants; this sample should provide sufficient data on feasibility and safety, and indicate estimates of potential effect size to calculate power for a future full trial.

#### Analysis

Using the Consolidated Standards of Reporting Trials (CONSORT) flow chart (Figure 1) (44) we will report on screened, included, and excluded patients, the number consented, and number of those with consent withdrawn. We will report on the number and proportion of children enrolled through prospective consent in comparison to consent to continue, timing of consent, and investigate the investigator-stated reasons if consent to continue was employed. Descriptive statistics will be used to compare demographics, clinical history and baseline clinical characteristics between the study arms (Supplementary Table 1), however statistical comparisons will not be undertaken. The estimate of the difference between the two study groups and the corresponding 95% confidence interval (CI) will be reported for outcomes relating to feasibility and clinical characteristics (Supplementary Tables 2 and 3). Quantile regression will be used to assess the comparison for continuous outcomes and the test of two proportions for binary outcomes. All analyses will be based on the intention-to-treat principle. All patients who underwent randomization and for whom consent is available are included in the ITT analysis. Protocol violations and major adverse events will be listed separately (Supplementary Table 4). A sensitivity analysis will be undertaken for primary and secondary outcomes, separating the standard care arm into two groups–those not receiving hydrocortisone, and those receiving hydrocortisone. Descriptive statistics only will be presented on the sensitivity analysis (Supplementary Table 5). In addition, we will provide graphs to describe temporal changes in pSOFA, physiological measures (heart rate, systolic blood pressure), serum lactate levels, use of fluid bolus volume (in ml/kg), and Vasopressor-Inotrope-Score (45) comparing the intervention group vs. controls, starting at time of randomization. Given the study sample, and that no stratification is performed, we will not perform other pre-planned subgroup analyses.

We anticipate that <30% of RESPOND PICU study patients will be co-enrolled into a concomitant study investigating early inotropes in sepsis, named RESPOND ED (ACTRN12619000828123). Analyses on RESPOND PICU will not be adjusted for co-enrolment in RESPOND ED given that both are pilot feasibility studies.

### Current Trial Status

RESPOND PICU commenced recruiting in June 2019 with a projected completion date of June 2021. Recruitment is live at the central study site Queensland Children's Hospital, at Gold Coast University Hospital, and started recently at Sydney Children‘s Hospital, Westmead Children‘s Hospital, Perth Children‘s Hospital, Australia, and Starship Children‘s Hospital, New Zealand.

### Significance

This worldwide first trial on metabolic resuscitation in paediatric sepsis will test the feasibility of an intervention showing promise of improving patient-centred outcomes and cost-effective health care delivery. Study sites include several paediatric hospitals across Australia and New Zealand, leveraging off of the ANZICS Paediatric Study Group network.

The study design is informed by the VITAMINS trial (24) and similar several recently published adult RCTs on metabolic resuscitation. While the optimal dosing of metabolic resuscitation, in particular of ascorbic acid, remains uncertain, weight-based dosing schedules were derived from adult studies, considering expert pediatric critical care pharmacist advice. Of note, we recently reviewed the literature on harm related to high dose ascorbic acid in children and identified that the safety of this intervention is very high even at doses superior to those used in this trial (46). Inclusion and exclusion criteria of this pilot study were adapted from adult trials towards pediatric populations, but it is acknowledged that criteria such as cardiomyopathy were not defined in detail to keep the study pragmatic. Limitations include lack of a dedicated hydrocortisone only arm, and hence pre-planned subgroup analyses on children treated with hydrocortisone, vs. controls without hydrocortisone, and vs. intervention with metabolic resuscitation, will be performed.

The interventions are low cost and are based on globally available drugs with excellent safety profiles_._ The study design is pragmatic, and findings will serve to inform the design of a fully powered trial on high dose ascorbic acid, thiamine, and hydrocortisone in children with septic shock.

## Ethics Statement

The studies involving human participants were reviewed and approved by Children's Health Queensland, Brisbane, HREC/18/QCHQ/49168. Written informed consent to participate in this study was provided by the participants' legal guardian/next of kin.

## Author Contributions

LS and RB were responsible for the initial protocol development. LS was responsible for subsequent protocol refinement with input from KG, RR, AH, MC, DL, DB, SE, MF, SG, MK, PS, and SR. KG supervised the REDCap setup and contributed to writing the statistical analysis plan. LS refined and developed subsequent manuscript drafts. All authors contributed to final manuscript preparation and approved final submission.

## Group Authorship: RESPOND PICU

Queensland Children's Hospital: A/Prof Luregn Schlapbach, Ms Amanda Harley, Dr Sainath Raman, Ms Roberta Ridolfi, Ms Natalie Sharp, A/Prof Kristen Gibbons, Ms Renate Le Marsnay, Ms Michele Cree, A/Prof Debbie Long

Austin Hospital and Monash University, Melbourne: Prof. Rinaldo Bellomo

Gold Coast University Hospital: Dr. Megan King, A/Prof. Shane George, Mr Nathan Goddard, Mr Kieran Owen

Perth Children‘s Hospital, Perth: Dr Simon Erickson, Ms Hannah Thomson

Sydney Children‘s Hospital, Sydney: Dr Puneet Singh, Ms Vicki Smith

The Children's Hospital at Westmead, Sydney: Dr Marino Festa, Dr Chong Tien Goh, Ms Gale Harper

Starship Children‘s Hospital, Auckland, NZ: Dr David Buckely, Dr John Beca, Ms Claire Sherring.

## Australian and New Zealand Intensive Care Society Paediatric Study Group (ANZICS PSG)

Anusha Ganeshalingam, Claire Sherring, Starship Children's Hospital, Auckland, New Zealand; Simon Erickson, Samantha Barr, Perth Children‘s Hospital, Perth, Australia; Sainath Raman, Debbie Long, Luregn Schlapbach (Past Chair), Kristen Gibbons (Vice Chair), Queensland Children's Hospital and The University of Queensland, Brisbane, Australia; Shane George, Gold Coast University Hospital; Puneet Singh, Vicky Smith, Sydney Children's Hospital, Randwick, Australia; Warwick Butt (Chair), Carmel Delzoppo, Johnny Millar (ANZPIC registry lead), Ben Gelbart, Royal Children's Hospital, Melbourne, Australia; Breanna Pellegrini (ANZPIC registry); Felix Oberender, Monash Children‘s Hospital, Melbourne, Australia; Subodh Ganu, Georgia Letton, Women's and Children's Hospital, Adelaide, Australia; Gail Harper, Marino Festa, Westmead Children's Hospital, Sydney, Australia.

## Conflict of Interest

The authors declare that the research was conducted in the absence of any commercial or financial relationships that could be construed as a potential conflict of interest.
