# Supplementary material for: Resuscitation in Paediatric Sepsis Using Metabolic Resuscitation–A Randomized Controlled Pilot Study in the Paediatric Intensive Care Unit (RESPOND PICU): Study Protocol and Analysis Plan
Source: Front Pediatr. 2021 Apr 30;9:663435. doi: 10.3389/fped.2021.663435 (PMC8142861; doi:10.3389/fped.2021.663435)
Supplement: Supplementary file 1 [file Data_Sheet_1.PDF]

Supplementary Materials for:

Resuscitation in Paediatric Sepsis Using Metabolic Resuscitation – A Pilot Randomized Controlled Pilot Study in the Paediatric Intensive Care Unit (RESPOND PICU): Study Protocol and Analysis Plan.

Luregn J Schlapbach[1,2], Kristen Gibbons[1], Roberta Ridolfi[1], Amanda Harley[1,3,4], Michele Cree[1,5], Debbie Long[1,6], David Buckley[7], Simon Erickson[8], Marino Festa[9,10], Shane George[1,3,11], Megan King[3], Puneet Singh[12], Sainath Raman[1], Rinaldo Bellomo[13], on behalf of the RESPOND PICU study investigators and the Australian and New Zealand Intensive Care Society Paediatric Study Group (ANZICS PSG)

[1] Child Health Research Centre, The University of Queensland, and Paediatric Intensive Care Unit, Queensland Children`s Hospital, Brisbane, QLD, Australia
[2] Pediatric and Neonatal Intensive Care Unit, and Children`s Research Center, University Children's Hospital Zurich, Zurich, Switzlerland
[3] Departments of Emergency Medicine and Children's Critical Care, Gold Coast University Hospital, Southport, Australia
[4] School of Nursing, Midwifery and Social Work, University of Queensland, QLD Australia
[5] Pharmacy Department, Queensland Children`s Hospital, Brisbane, QLD, Australia
[6] School of Nursing, Centre for Healthcare Transformation, Queensland University of Technology, Brisbane, QLD, Australia
[7] Paediatric Intensive Care Unit, Starship Children's Hospital, Auckland, New Zealand
[8] Paediatric Critical Care Unit, Perth Children`s Hospital, Perth, Australia
[9] Paediatric Intensive Care Unit, Children's Hospital Westmead, Sydney, Australia
[10] Kids Critical Care Research Group, Kids Research, Sydney Children's Hospitals Network, Sydney, Australia
[11] School of Medicine and Menzies Health Institute Queensland, Griffith University, Southport, Australia
[12] Paediatric Intensive Care Unit, Sydney Children's Hospital, Sydney, Australia
[13] Intensive Care Research, Austin Hospital and Monash University, Melbourne, Australia

Corresponding author:
Prof. Luregn Schlapbach, MD, PhD
Head, Pediatric and Neonatal Intensive Care Unit
University Children's Hospital Zurich – Eleonore Foundation
Steinwiesstrasse 75
CH-8032 Zurich Switzerland
phone +41 44 266 71 11
email: l.schlapbach@uq.edu.au

List of supplementary Materials:

1. **Statistical analysis code.**
2. **Supplementary Mock Table 1: Baseline characteristics of infants enrolled in the RESPOND PICU trial**
3. **Supplementary Mock Table 2. Feasibility outcomes per intention-to-treat analysis, with sensitivity analyses by site.**
4. **Supplementary Mock Table 3. Primary and secondary clinical outcomes per intention-to-treat analysis**
5. **Supplementary Mock Table 4. Protocol violations and major adverse events**
6. **Supplementary Mock Table 5. Primary and secondary clinical outcomes per sensitivity analysis**
7. **Supplementary Mock Table 6. Primary and secondary clinical outcomes for subgroup of patients with septic shock and acute lung injury.**

Statistical analysis code.

The RESPOND PICU Pilot study dataset is contained within a single REDCap database containing records on all screened patients from the RESPOND ED and RESPOND PICU studies (data fields include date of screening, inclusion criteria, exclusion criteria, eligibility status, informed consent process, withdrawal of consent), and all consented patients (randomisation details, demographics, clinical history, baseline assessment, treatments and management, outcomes, biobanking, 6-month follow up, adverse events and protocol deviations).

The RESPOND PICU study dataset will be exported from REDCap using the in-built functionality into Stata format; a Stata compatible dataset in comma-separated value (CSV) format (.csv) and Stata do-file (.do) is generated. The study dataset contains one row per screened patient per repeating event.

The code is broken into four sections, two of which are relevant for this protocol:

Part A: Transformation of primary study dataset and calculation of outcomes.

Part D: Analysis of patients enrolled in the PICU study ("RESPOND PICU Data Analysis.do")

The code has been uploaded to github and is available under the following link:

https://github.com/kgibbons44/RESPONDPilotAnalysis

Supplementary Mock Table 1: Baseline characteristics of infants enrolled in the RESPOND PICU trial

**Characteristic**	**Standard Care N=xx**	**Metabolic Resuscitation N=xx**
**Age at randomisation (months)** *median (IQR)*		
**Weight (kg)** *median (IQR)*		
**Female sex** *n (%)*		
**Ethnicity**		
Caucasian *n (%)*		
Aboriginal/Torres Strait Islander *n (%)*		
Asian *n (%)*		
Maori/Pacific Islander *n (%)*		
Mixed/Other *n (%)*		
**Chronic disease** *n (%)*		
Congenital malformation *n (%)*		
Asthma *n (%)*		
Congenital heart defect *n (%)*		
Oncologic disease *n (%)*		
Cerebral palsy/severe encephalopathy *n (%)*		
Metabolic disorder *n (%)*		
Primary immunodeficiency *n (%)*		
Syndrome/genetic disorder *n (%)*		
Other *n (%)*		
**Baseline POPC** *median (IQR)* *		
**Baseline Functional Status Score** *median (IQR)*		
**Observations at baseline**		
Heart rate *median (IQR)*		
Respiratory rate *median (IQR)*		
Systolic blood pressure *median (IQR)*		
Mean blood pressure *median (IQR)*		
Diastolic blood pressure *median (IQR)*		
Temperature *median (IQR)*		
SpO_2 *median (IQR)*		
FiO_2 at time of SpO_2 *median (IQR)*		
PaO_2 / FiO_2 ratio *median (IQR)*		
High-flow nasal cannulae support *n (%)*		
Non-invasive respiratory support *n (%)*		
Invasive respiratory support *n (%)*		
Capillary refill time		
Not measured *n (%)*		
<2 sec *n (%)*		
2-5 sec *n (%)*		

Characteristic	Standard Care N=xx	Metabolic Resuscitation N=xx
>5 sec *n (%)*		
Glasgow Coma Score *median (IQR)*		
Dilated unresponsive pupils *n (%)*		
**Laboratory**		
pH *median (IQR)*		
Base excess [mmol/l] *median (IQR)*		
paO_2 [mmHg] *median (IQR)*		
pCO_2 [mmHg] *median (IQR)*		
Lactate [mmol/l] *median (IQR)*		
Glucose [mmol/l] *median (IQR)*		
Sodium [mmol/l] *median (IQR)*		
Chloride [mmol/l] *median (IQR)*		
Creatinine [µmol/l] *median (IQR)*		
Bilirubin [µmol/l] *median (IQR)*		
Alanine aminotransferase level [U/L] *median (IQR)*		
International Normalized Ratio *median (IQR)*		
Fibrinogen [g/L] *median (IQR)*		
Platelets [$*10^3$/µL] *median (IQR)*		
White Cell Count [$*10^3$/µL] *median (IQR)*		
Absolute Neutrophil Count [$*10^3$/µL] *median (IQR)*		
Haemoglobin [g/L] *median (IQR)*		
C-reactive protein [mg/L] *median (IQR)*		
**Organ dysfunction score**		
pSOFA *median (IQR)*		
PELOD-2 *median (IQR)*		
**Treatment prior to randomization**		
Total amount of fluid boluses received within the past 4 hours [ml/kg] *median (IQR)*		
Time since intravenous antibiotics were started [hours] *median (IQR)*		
Vasoactive inotrope score *median (IQR)*		
Intravenous steroids for septic shock *n (%)*		
Renal replacement therapy *n (%)*		
Extracorporeal membrane oxygenation *n (%)*		

IQR, interquartile range; pSOFA, pediatric Sequential Organ Failure Assessment; PELOD-2, Pediatric Logistic Organ Dysfunction score-2; POPC, Pediatric Overall Performance Category

[*] the POPC category “good” (healthy, alert, and capable of normal age-appropriate activities of daily life; medical and physical problems do not interfere with normal activity) was further divided into “good/normal” (no medical conditions), and b) “functionally normal” (requires medication and medical input, normal intellectually and physically, able to do activities without restriction), thus resulting in a 7-point POPC scale.

Supplementary Mock Table 2a. Feasibility outcomes per intention-to-treat analysis for all sites

Outcome	Standard Care N=xx	Metabolic resuscitation N=xx	Estimate of Difference (95% CI)
Time from screening to randomisation [min] *median (IQR)*			
Time from PICU admission to randomisation [min] *median (IQR)*			
Time from randomisation to commencement of metabolic resuscitation [min] *median (IQR)*			
Hydrocortisone received after randomisation *n (%)*			
Cumulative hydrocortisone dose after randomisation [mg/kg] *median IQR*			
Ascorbic acid received after randomisation *n (%)*			
Cumulative ascorbic acid dose after randomisation [mg/kg] *median IQR*			
Thiamine received after randomisation *n (%)*			
Cumulative thiamine dose after randomisation [mg/kg] *median IQR*			
Time from inotrope infusion commencement to randomisation [min] *median (IQR)*			

CI, confidence interval; IQR, interquartile range; PICU, paediatric intensive care unit

Supplementary Mock Table 2b. Feasibility outcomes per intention-to-treat analysis for the lead site

**Outcome**	**Standard Care N=xx**	**Metabolic resuscitation N=xx**	**Estimate of Difference (95% CI)**
Time from screening to randomisation [min] *median (IQR)*			
Time from PICU admission to randomisation [min] *median (IQR)*			
Time from randomisation to commencement of metabolic resuscitation [min] *median (IQR)*			
Hydrocortisone received after randomisation *n (%)*			
Cumulative hydrocortisone dose after randomisation [mg/kg] *median IQR*			
Ascorbic acid received after randomisation *n (%)*			
Cumulative ascorbic acid dose after randomisation [mg/kg] *median IQR*			
Thiamine received after randomisation *n (%)*			
Cumulative thiamine dose after randomisation [mg/kg] *median IQR*			
Time from inotrope infusion commencement to randomisation [min] *median (IQR)*			

CI, confidence interval; IQR, interquartile range; PICU, paediatric intensive care unit

Supplementary Mock Table 2c. Feasibility outcomes per intention-to-treat analysis excluding the lead site

**Outcome**	**Standard Care N=xx**	**Metabolic resuscitation N=xx**	**Estimate of Difference (95% CI)**
Time from screening to randomisation [min] *median (IQR)*			
Time from PICU admission to randomisation [min] *median (IQR)*			
Time from randomisation to commencement of metabolic resuscitation [min] *median (IQR)*			
Hydrocortisone received after randomisation *n (%)*			
Cumulative hydrocortisone dose after randomisation [mg/kg] *median IQR*			
Ascorbic acid received after randomisation *n (%)*			
Cumulative ascorbic acid dose after randomisation [mg/kg] *median IQR*			
Thiamine received after randomisation *n (%)*			
Cumulative thiamine dose after randomisation [mg/kg] *median IQR*			
Time from inotrope infusion commencement to randomisation [min] *median (IQR)*			

CI, confidence interval; IQR, interquartile range; PICU, paediatric intensive care unit

Supplementary Mock Table 3. Primary and secondary clinical outcomes per intention-to-treat analysis

Outcome	Standard Care N=xx	Metabolic resuscitation N=xx	Estimate of Difference (95% CI)
**Primary clinical outcome**			
Survival free of organ dysfunction* censored at 28 days *median (IQR)*			
**Secondary clinical outcomes**			
Survival free of inotrope support at 7 days *median (IQR)*			
Survival free of multiorgan dysfunction** at 7 days *median (IQR)*			
Survival free of Acute Kidney Injury*** at 28 days *median (IQR)*			
28-day mortality *n (%)*			
Survival free of PICU censored at 28 days *median (IQR)*			
Length of stay in PICU *median (IQR)*			
Length of stay in hospital *median (IQR)*			
POPC**** at 28 days *median (IQR)*			
Change in POPC**** from baseline *median (IQR)*			
Functional Status Score at 28 days *median (IQR)*			
Change in Functional Status Score from baseline *median (IQR)*			
**Proxy measures of intervention efficacy**			
Lactate <2mmol/l by 6 hours post enrolment *n (%)*			
Lactate <2mmol/l by 12 hours post enrolment *n (%)*			
Lactate <2mmol/l by 24 hours post enrolment *n (%)*			
Time to reversal of tachycardia censored at 24 hours [hours] *median (IQR)*			
Time to shock reversal censored at 28 days [hours] *median (IQR)*			

CI, confidence interval; IQR, interquartile range; PICU, paediatric intensive care unit; POPC Pediatric Overall Performance Category

* as measured by pediatric Sequential Organ Failure Assessment (pSOFA) score

** multi-organ dysfunction is defined as >1 organ with a pSOFA subscore of >0

*** Acute Kidney Injury (AKI) will be assessed using serum creatinine levels to classify according to Kidney Disease: Improving Global Outcomes (KDIGO) criteria. Because no baseline creatinine values were available, we applied the age-specific thresholds used in PELOD-2 to define the presumed baseline creatinine values. KDIGO Stage 1 was defined as an increase in creatinine to 1.5 to 1.9 times the presumed baseline; Stage 2 as an increase 2.0 to 2.9 times; and KDIGO 3 as an increase ≥3.0 baseline and/or the use of renal replacement therapy.

**** the POPC category "good" (healthy, alert, and capable of normal age-appropriate activities of daily life; medical and physical problems do not interfere with normal activity) was further divided into "good/normal" (no medical conditions), and b) "functionally normal" (requires medication and medical input, normal intellectually and physically, able to do activities without restriction), thus resulting in a 7-point POPC scale.

Supplementary Mock Table 4. Protocol violations and major adverse events

**Variable**	**Standard Care N=xx**	**Metabolic resuscitation N=xx**	**Estimate of Difference (95% CI)**
**Protocol violations**	N=xx	N=xx	
Any protocol deviation *n (%)*			
Consultant or investigator initiated withdrawal from the study prior to the finalisation of informed consent *n (%)*			
Time taken to obtain written informed consent exceeded 72 hours *n (%)*			
Written informed consent obtained but no study data collected *n (%)*			
Patient randomised but did not meet study specified inclusion/exclusion criteria for enrolment in the study *n (%)*			
Patient randomised to an incorrect strata *n (%)*			
Patient randomised but did not receive/commence on any study treatment(s) *n (%)*			
First treatment received/commenced is not the same as the randomised allocation *n (%)*			
Study treatment not delivered according to protocol *n (%)*			
Other			
**Major adverse events**	N=xx	N=xx	
Any adverse event *n (%)*			
Death *n (%)*			
Cardiopulmonary arrest *n (%)*			
Extracorporeal membrane oxygenation *n (%)*			
Amputation *n (%)*			
Limb ischemia *n (%)*			
Extravasation injury *n (%)*			
Hypertension *n (%)*			
Arrhythmia *n (%)*			
Hyperglycemia *n (%)*			

Abdominal compartment syndrome *n (%)*			
Pulmonary oedema *n (%)*			
Confirmed hospital-acquired infection *n (%)*			
Other *n (%)*			
Relatedness of the AE with the study intervention			
Not related *n (%)*			
Unlikely *n (%)*			
Possibly *n (%)*			
Probably *n (%)*			
Definitely *n (%)*			

Supplementary Mock Table 5. Primary and secondary clinical outcomes per sensitivity analysis

Outcome	Standard Care without Hydrocortisone N=xx	Standard Care with Hydrocortisone N=xx	Metabolic resuscitation N=xx
**Primary clinical outcome**			
Survival free of organ dysfunction* censored at 28 days *median (IQR)*			
**Secondary clinical outcomes**			
Survival free of inotropic support at 7 days *n (%)*			
Survival free of multiorgan dysfunction** at 7 days *n (%)*			
Survival free of Acute Kidney Injury*** at 28 days *median (IQR)*			
28-day mortality *n (%)*			
Survival free of PICU censored at 28 days *median (IQR)*			
Length of stay in PICU *median (IQR)*			
Length of stay in hospital *median (IQR)*			
POPC**** at 28 days *median (IQR)*			
Change in POPC**** from baseline *median (IQR)*			
Functional Status Score at 28 days *median (IQR)*			
Change in Functional Status Score from baseline *median (IQR)*			
**Proxy measures of intervention efficacy**			
Lactate <2mmol/l by 6 hours post enrolment *n (%)*			
Lactate <2mmol/l by 12 hours post enrolment *n (%)*			
Lactate <2mmol/l by 24 hours post enrolment *n (%)*			
Time to reversal of tachycardia censored at 24 hours [hours] *median (IQR)*			
Time to shock reversal censored at 28 days [hours] *median (IQR)*			

IQR, interquartile range; PICU, paediatric intensive care unit; POPC Pediatric Overall Performance Category
* as measured by pediatric Sequential Organ Failure Assessment (pSOFA) score

[**]multi-organ dysfunction is defined as >1 organ with a pSOFA subscore of >0
[***] Acute Kidney Injury (AKI) will be assessed using serum creatinine levels to classify according to Kidney Disease: Improving Global Outcomes (KDIGO) criteria. Because no baseline creatinine values were available, we applied the age-specific thresholds used in PELOD-2 to define the presumed baseline creatinine values. KDIGO Stage 1 was defined as an increase in creatinine to 1.5 to 1.9 times the presumed baseline; Stage 2 as an increase 2.0 to 2.9 times; and KDIGO 3 as an increase ≥3.0 baseline and/or the use of renal replacement therapy.
[****] the POPC category "good" (healthy, alert, and capable of normal age-appropriate activities of daily life; medical and physical problems do not interfere with normal activity) was further divided into "good/normal" (no medical conditions), and b) "functionally normal" (requires medication and medical input, normal intellectually and physically, able to do activities without restriction), thus resulting in a 7-point POPC scale.

Supplementary Mock Table 6. Primary and secondary clinical outcomes for subgroup of patients with septic shock and acute lung injury (ALI). ALI was defined as study patients with a pediatric Sequential Organ Failure Assessment (pSOFA) respiratory subscore of ≥2 at randomization.

**Outcome**	**Standard Care N=xx**	**Metabolic resuscitation N=xx**
**Primary clinical outcome**		
Survival free of organ dysfunction* censored at 28 days *median (IQR)*		
**Secondary clinical outcomes**		
Survival free of inotropic support at 7 days *n (%)*		
Survival free of multiorgan dysfunction** at 7 days *n (%)*		
Survival free of Acute Kidney Injury*** at 28 days *median (IQR)*		
28-day mortality *n (%)*		
Survival free of PICU censored at 28 days *median (IQR)*		
Length of stay in PICU *median (IQR)*		
Length of stay in hospital *median (IQR)*		
POPC**** at 28 days *median (IQR)*		
Change in POPC**** from baseline *median (IQR)*		
Functional Status Score at 28 days *median (IQR)*		
Change in Functional Status Score from baseline *median (IQR)*		
**Proxy measures of intervention efficacy**		
Lactate <2mmol/l by 6 hours post enrolment *n (%)*		
Lactate <2mmol/l by 12 hours post enrolment *n (%)*		
Lactate <2mmol/l by 24 hours post enrolment *n (%)*		
Time to reversal of tachycardia censored at 24 hours [hours] *median (IQR)*		
Time to shock reversal censored at 28 days [hours] *median (IQR)*		

IQR, interquartile range; PICU, paediatric intensive care unit; POPC Pediatric Overall Performance Category
[*] as measured by pediatric Sequential Organ Failure Assessment (pSOFA) score
[**]multi-organ dysfunction is defined as >1 organ with a pSOFA subscore of >0
[***] Acute Kidney Injury (AKI) will be assessed using serum creatinine levels to classify according to Kidney Disease: Improving Global Outcomes (KDIGO) criteria. Because no baseline creatinine values were available, we applied the age-specific thresholds used in PELOD-2 to define the presumed baseline creatinine values. KDIGO Stage 1 was defined as an increase in creatinine to 1.5 to 1.9 times the presumed baseline; Stage 2 as an increase 2.0 to 2.9 times; and KDIGO 3 as an increase ≥3.0 baseline and/or the use of renal replacement therapy.
[****] the POPC category "good" (healthy, alert, and capable of normal age-appropriate activities of daily life; medical and physical problems do not interfere with normal activity) was further divided into "good/normal" (no medical conditions), and b) "functionally normal" (requires medication and medical input, normal intellectually and physically, able to do activities without restriction), thus resulting in a 7-point POPC scale.
